# Transcatheter Edge-to-Edge Repair for Acute Mitral Regurgitation due to Postinfarction Papillary Muscle Rupture

**DOI:** 10.1016/j.jscai.2022.100431

**Published:** 2022-08-09

**Authors:** Chih-Wei Chang, Steven Romero, Matthew J. Price

**Affiliations:** Division of Cardiology, Scripps Clinic, San Diego, California

**Keywords:** acute mitral regurgitation, MitraClip, papillary muscle rupture, transcatheter edge-to-edge repair

## Abstract

**Background:**

Early case reports have suggested that transcatheter edge-to-edge repair (TEER) with the MitraClip for the treatment of acute mitral regurgitation (MR) due to post-infarction papillary muscle rupture (PMR) may be an alternative for patients at prohibitive surgical risk. However, data on consecutive patients treated for this condition is lacking.

**Methods:**

To define the procedural characteristics, medication use, hemodynamic parameters, and imaging data, we present 5 consecutive patients with acute MR from postinfarction PMR treated with TEER at Scripps Memorial Hospital La Jolla between June 2018 and November 2020.

**Results:**

Successful reduction of MR and improved hemodynamics were achieved in all cases. Despite the procedural success, only 1 of the 5 patients survived until hospital discharge.

**Conclusion:**

TEER may be an effective treatment for acute MR due to postinfarction PMR to reduce MR and improve hemodynamics. Survival to discharge was infrequent, suggesting that TEER may be appropriate only in selected patients.

## Highlights

• Postinfarction papillary muscle rupture treated with transcatheter edge-to-edge repair
• Acute procedural success was achieved, with improved hemodynamics
• Survival to discharge was uncommon
• Age (young), STS risk (low), and mechanical support (none) were prognostic
• The cause of death was sequelae of early hemodynamic decompensation

## Central Illustration

**Figure:**
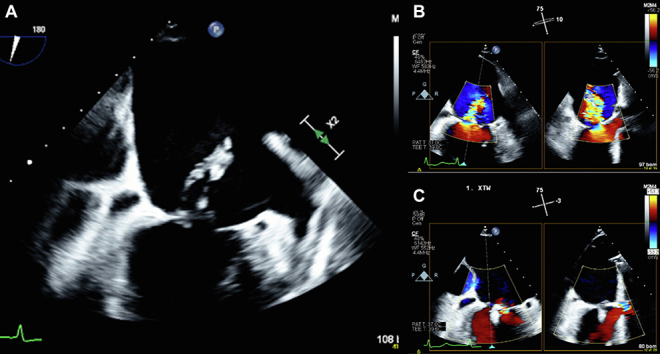
Postinfarction papillary muscle rupture with acute mitral regurgitation treated with transcatheter edge-to-edge repair.

## Introduction

Acute mitral regurgitation (MR) due to papillary muscle rupture (PMR) occurs in 2.3% of patients with acute myocardial infarction (MI).^1^ Postinfarction PMR typically occurs within 5 days after MI and carries a mortality rate of 80% during the first week without emergency surgical interventions.^2^ Although surgery is the standard of care, more than half of patients are excluded because of prohibitive operative risk, and among those who undergo surgery, the operative mortality rate is as high as 40%.^3^^,^^4^ Transcatheter edge-to-edge repair (TEER) with MitraClips (Abbott Vascular) is an established therapy for reducing degenerative and functional MR.^5^^,^^6^ Although early case reports have demonstrated that TEER for the treatment of postinfarction acute MR due to PMR is a feasible alternative for patients at prohibitive surgical risk, data on consecutive patients treated for this condition are lacking,^1^, ^2^, ^3^, ^4^^,^^7^, ^8^, ^9^, ^10^ and further characterization of patients’ clinical profiles, procedural characteristics, hemodynamic parameters, and outcomes is needed. For this purpose, we present 5 consecutive patients who underwent TEER for the treatment of acute MR due to PMR as a mechanical complication of MI and provide a comprehensive review of the existing literature for this management approach.

## Case presentation

### Case 1

**Figure 1:**
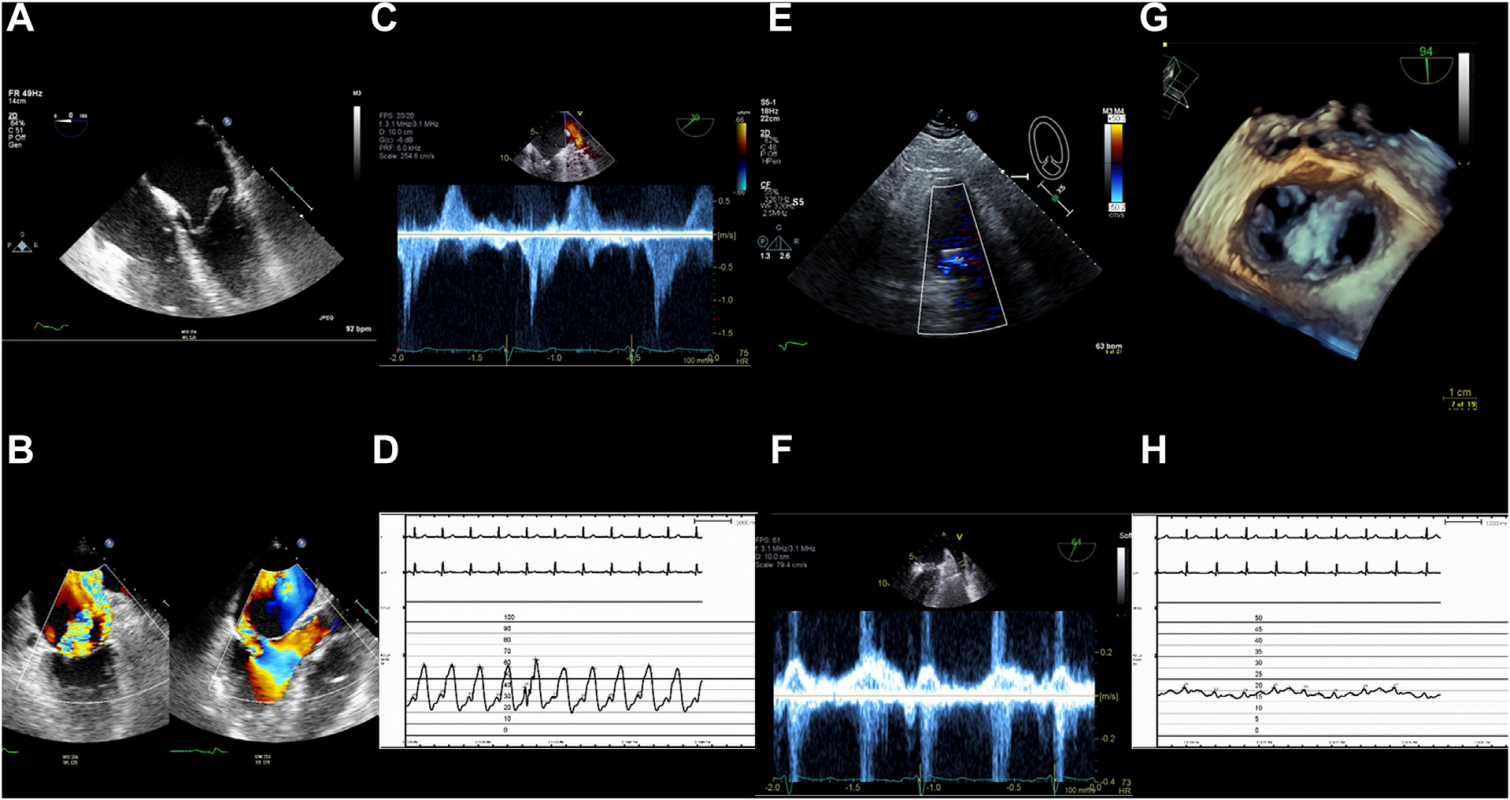
**Case 1 summary.** (**A**) TEE: complete rupture of the anterolateral papillary muscle. (**B**) TEE: severe mitral regurgitation across A2-P2. (**C**) TEE: pulmonary vein systolic flow reversal. (**D**) Elevated left atrial V wave pressure of 62 mm Hg. (**E**) Transthoracic echocardiogram: postprocedural mild mitral regurgitation. (**F**) TEE: postprocedural normalized pulmonary vein systolic flow. (**G**) TEE: postprocedural multiplanar reconstruction. (**H**) Reduced left atrial V wave pressure of 20 mm Hg. TEE, transesophageal echocardiogram.

A 56-year-old man with morbid obesity presented with a 4-day history of chest discomfort and was found to have cardiogenic shock due to an inferior ST-segment elevation MI (Figure 1). Coronary angiography demonstrated complete occlusion of the midright coronary artery, and, therefore, an intra-aortic balloon pump (IABP) was placed. A transesophageal echocardiogram demonstrated moderately reduced right ventricular systolic function. Given his profound hypoxia, right ventricular failure, and cardiogenic shock with pulmonary edema, he was placed on venoarterial extracorporeal membrane oxygenation (VA-ECMO) in addition to the IABP.

Given his right ventricular failure; persistent lactic acidosis; profound hypoxia; obesity, with a body mass index of 36 kg/m^2^; and a Society of Thoracic Surgeons Predicted Risk of Mortality (STS-PROM) score of 19%, he was deemed to be at prohibitive surgical risk, and he underwent TEER for complete rupture of the anterolateral papillary muscle and severe MR. Two MitraClip NT devices were implanted at A2-P2, which resulted in a reduction of the left atrial V wave from 62 to 20 mm Hg, normalization of pulmonary venous systolic flow, and moderate MR.

The patient was decannulated on postprocedural day 7. The postprocedural course was complicated by a hematoma due to VA-ECMO decannulation, requiring vascular repair; an upper gastrointestinal bleed; and renal failure, requiring hemodialysis. Given the improvement in hemodynamics and the prolonged period between the inciting inferior ST-segment elevation MI, right coronary artery revascularization or mitral valve surgery was not performed.

The patient was discharged on hospital day 41. A transthoracic echocardiogram (TTE) at the time of discharge demonstrated mild MR and a pulmonary artery systolic pressure of 36 mm Hg. At 1 month of follow-up, he remained in New York Heart Association functional class I.

### Case 2

**Figure 2:**
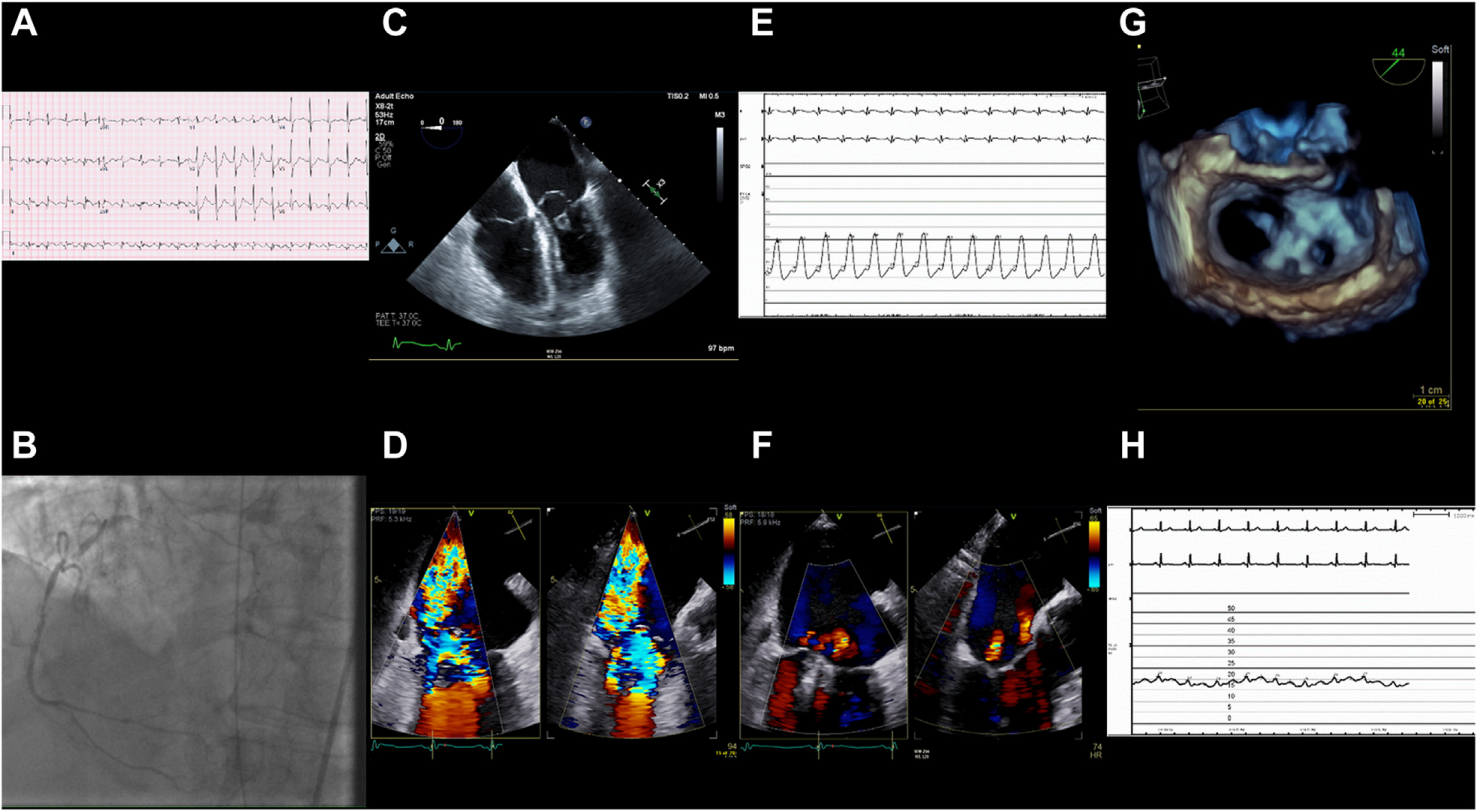
**Case 2 summary.** (**A**) Electrocardiogram: inferior ST-elevation myocardial infarction. (**B**) Coronary angiography: severe stenosis in the proximal and midright coronary arteries. (**C**) TEE: complete rupture of the posteromedial papillary muscle. (**D**) TEE: severe mitral regurgitation extending from A3-P3 to A2-P2. (**E**) Elevated left atrial V wave pressure of 52 mm Hg. (**F**) TEE: postprocedural mild mitral regurgitation. (**G**) TEE: postprocedural multiplanar reconstruction. (**H**) Reduced left atrial V wave pressure of 24 mm Hg. TEE, transesophageal echocardiogram.

A 68-year-old man with obesity, diabetes, hypertension, and active smoking presented with a 1-week history of chest pain with cardiogenic shock due to an inferior ST-segment elevation MI (Figure 2). An IABP was placed, and the right coronary artery was revascularized. The patient was treated with inotropes and vasopressors. Additionally, he was started on empiric antibiotics for possible aspiration given his leukocytosis, fever, and persistently elevated procalcitonin levels.

On hospitalization day 2, a TTE revealed a dilated right ventricle, with moderately decreased right ventricular systolic function. The TEE revealed a completely ruptured posteromedial papillary muscle. Given his prohibitive surgical risk from the cardiogenic shock due to multiple vasoactive infusions, an ongoing infection, obesity with a body mass index of 31 kg/m^2^, coagulopathy in the setting of dual antiplatelet therapy and thrombocytopenia, with an STS-PROM score of 28%, he underwent TEER for severe MR due to complete rupture of the posteromedial papillary muscle with flail A2, A3, and P3 scallops. He had 3 XTR devices implanted across the A3-P3 and A2-P2 scallops, resulting in a left atrial V wave reduction from 52 to 24 mm Hg and mild MR.

The patient’s postprocedural course was complicated by the need for hemodialysis, continued vasopressors, persistent fevers, and a lack of cognitive response despite sedation interruption. On postprocedural day 7, the patient’s hemodynamic and respiratory status deteriorated; a TTE revealed moderate-to-severe MR and single-leaflet device attachment of the most medial clip. Given the patient’s lack of cognitive function, the patient was transitioned to comfort care, and he expired on hospital day 8.

### Case 3

**Figure 3:**
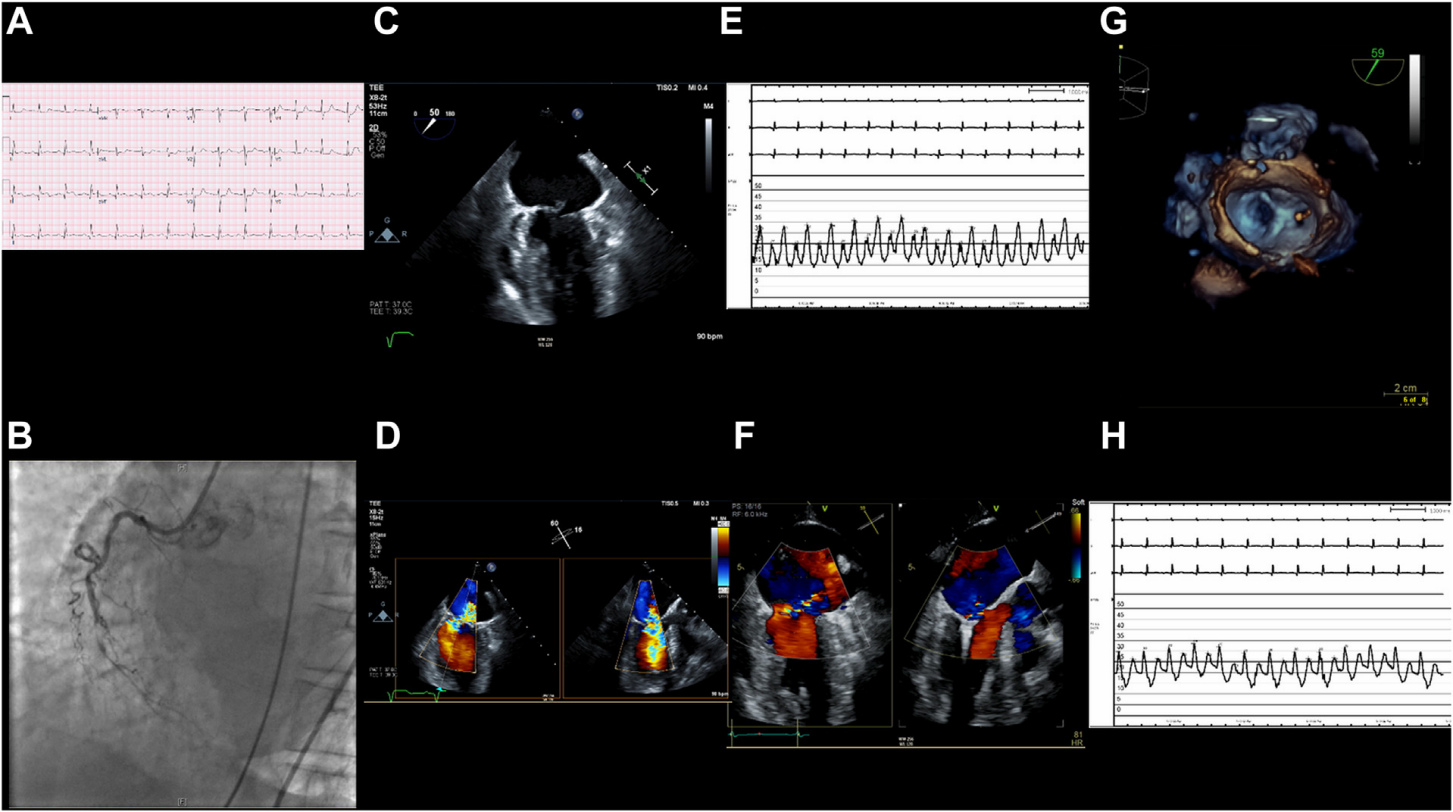
**Case 3 summary.** (**A**) Electrocardiogram: inferior ST-elevation myocardial infarction. (**B**) Coronary angiography: subtotal occlusion of the midright coronary artery. (**C**) TEE: partial rupture of the posteromedial papillary muscle. (**D**) TEE: severe mitral regurgitation extending from A3-P3 to A2-P2. (**E**) Elevated left atrial V wave pressure of 34 mm Hg. (**F**) TEE: postprocedural trace mitral regurgitation. (**G**) TEE: postprocedural multiplanar reconstruction. (**H**) Reduced left atrial V wave pressure of 26 mm Hg. TEE, transesophageal echocardiogram.

An 81-year-old woman with hypertension, dementia, chronic obstructive pulmonary disease, and chronic renal disease presented with chest discomfort with cardiogenic shock due to an inferior ST-segment elevation MI (Figure 3). Catheterization revealed subtotal occlusion of the midright coronary artery and severe MR. An IABP was placed. Given her prohibitive surgical risk and an STS-PROM score of 23%, she underwent TEER for the severe MR due to partial rupture of the posteromedial papillary muscle with flail P2 and P3 scallops and bileaflet prolapse. She had an XTR device implanted at A2-P2, and an NTR device implanted at A3-P3 resulted in a reduction of the left atrial V wave from 34 to 26 mm Hg and trace MR.

On the first day after the procedure, the patient was weaned off vasoactive medications, and the IABP was removed. However, her hospital course was then complicated by septic shock and worsening renal function. The family declined hemodialysis, and the patient was transitioned to comfort care. She subsequently expired.

### Case 4

**Figure 4:**
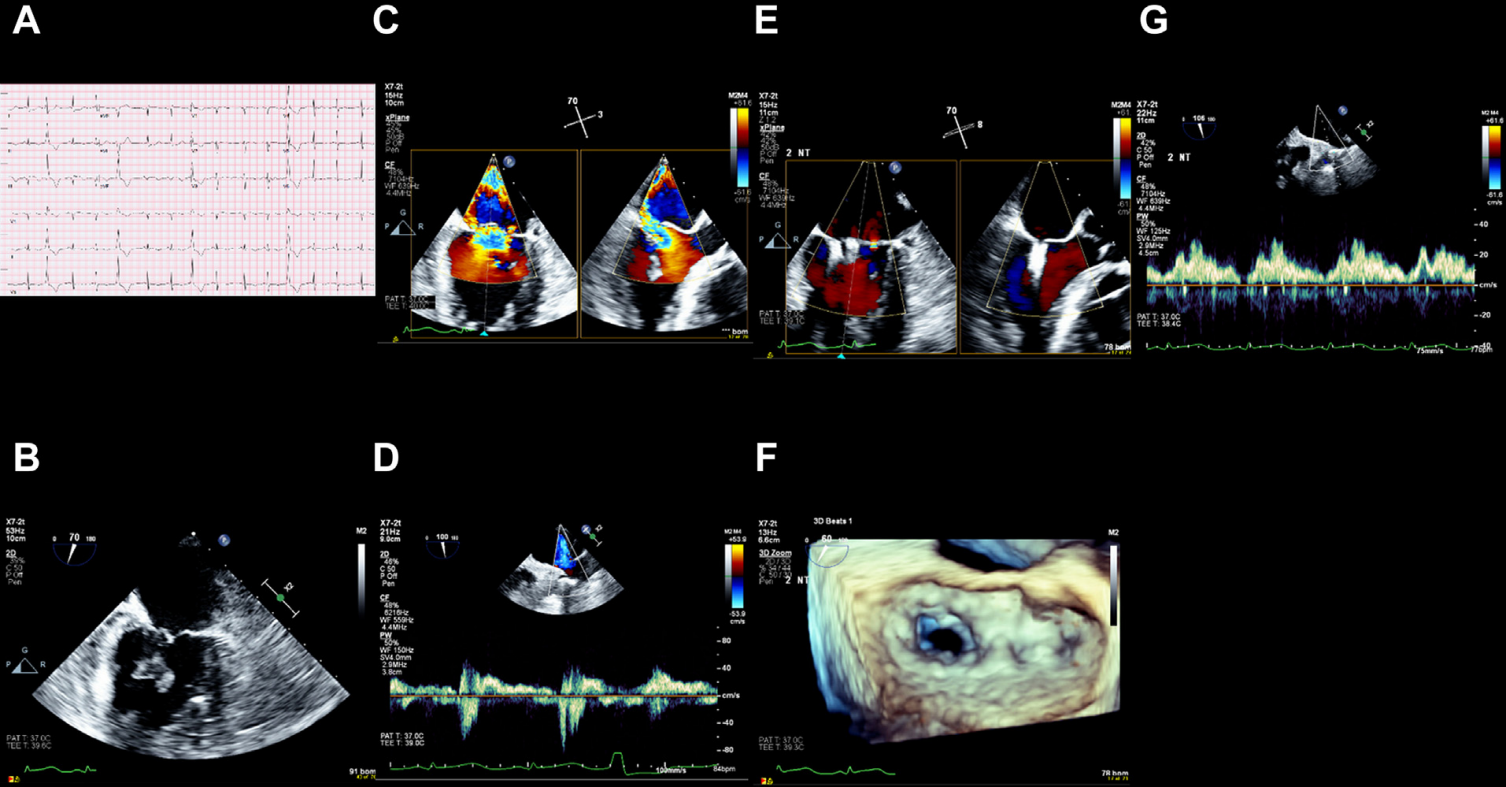
**Case 4 summary.** (**A**) Electrocardiogram: inferior ST-elevation myocardial infarction. (**B**) TEE: partial rupture of the posteromedial papillary muscle. (**C**) TEE: severe mitral regurgitation. (**D**) TEE: pulmonary vein systolic flow reversal. (**E**) TEE: postprocedural trace mitral regurgitation. (**F**) TEE: postprocedural multiplanar reconstruction. (**G**) TEE: postprocedural normalized pulmonary vein systolic flow. TEE, transesophageal echocardiogram.

An 86-year-old woman who was initially admitted for a gastrointestinal bleed due to a large duodenal ulcer unamenable to endoscopic treatment had chest discomfort due to an inferior ST-segment elevation MI (Figure 4). She was managed medically, but she developed cardiogenic shock 48 hours later. An IABP was placed. Given her prohibitive surgical risk and an STS-PROM score of 25%, she underwent TEER for severe MR due to partial rupture of the posteromedial papillary muscle. An XTW device was implanted at the medial aspect of A2-P2, and an NT device was implanted medial to the first clip, resulting in normalization of pulmonary venous systolic flow and trace MR. A large right-to-left shunt was closed using a 10-mm Amplatzer septal occluder.

The patient’s hemodynamics improved after the procedure. However, she remained on inotropes, vasopressors, IABP, and antibiotics for an *Escherichia coli* urinary tract infection. The patient became anuric, requiring renal replacement therapy. Given the patient’s goals of care, she was transitioned to comfort care, and she expired on postprocedural day 2.

### Case 5

**Figure 5:**
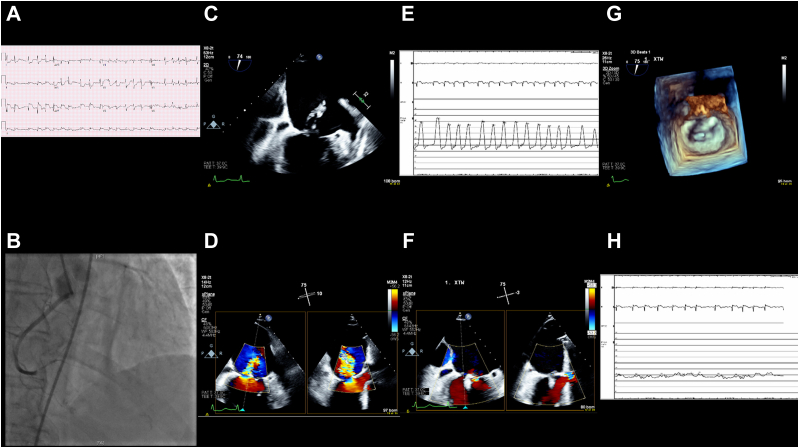
**Case 5 summary.** (**A**) Electrocardiogram: inferior-posterior, ST-elevation myocardial infarction. (**B**) Coronary angiography: complete occlusion of the proximal posterior descending artery and subtotal occlusion of the proximal posterolateral branch. (**C**) TEE: complete rupture of the posteromedial papillary muscle. (**D**) TEE: severe mitral regurgitation. (**E**) Elevated left atrial V wave pressure of 39 mm Hg. (**F**) TEE: postprocedural trace mitral regurgitation. (**G**) TEE: postprocedural multiplanar reconstruction. (**H**) Reduced left atrial V wave pressure of 15 mm Hg. TEE, transesophageal echocardiogram.

An 84-year-old woman with hypertension and atrial fibrillation presented with 2-day history of nausea and vomiting and was found to have cardiogenic shock due to an inferior-posterior ST-segment elevation MI (Figure 5). After resuscitation for cardiac arrest during cardiac catheterization, her proximal posterior descending artery, with an occlusion of 100%, and proximal posterolateral branch, with an occlusion of 90%, were revascularized, and an IABP was inserted. Given her prohibitive surgical risk and an STS-PROM score of 66%, she underwent TEER for severe MR due to complete rupture of the posteromedial papillary muscle. She had an XTW device implanted at A2-P2 (Central Illustration), which resulted in a reduction of the left atrial V wave from 39 to 15 mm Hg and trace MR. A significant right-to-left shunt was closed using a 20-mm Gore Cardioform septal occlude.

**Central Illustration:**
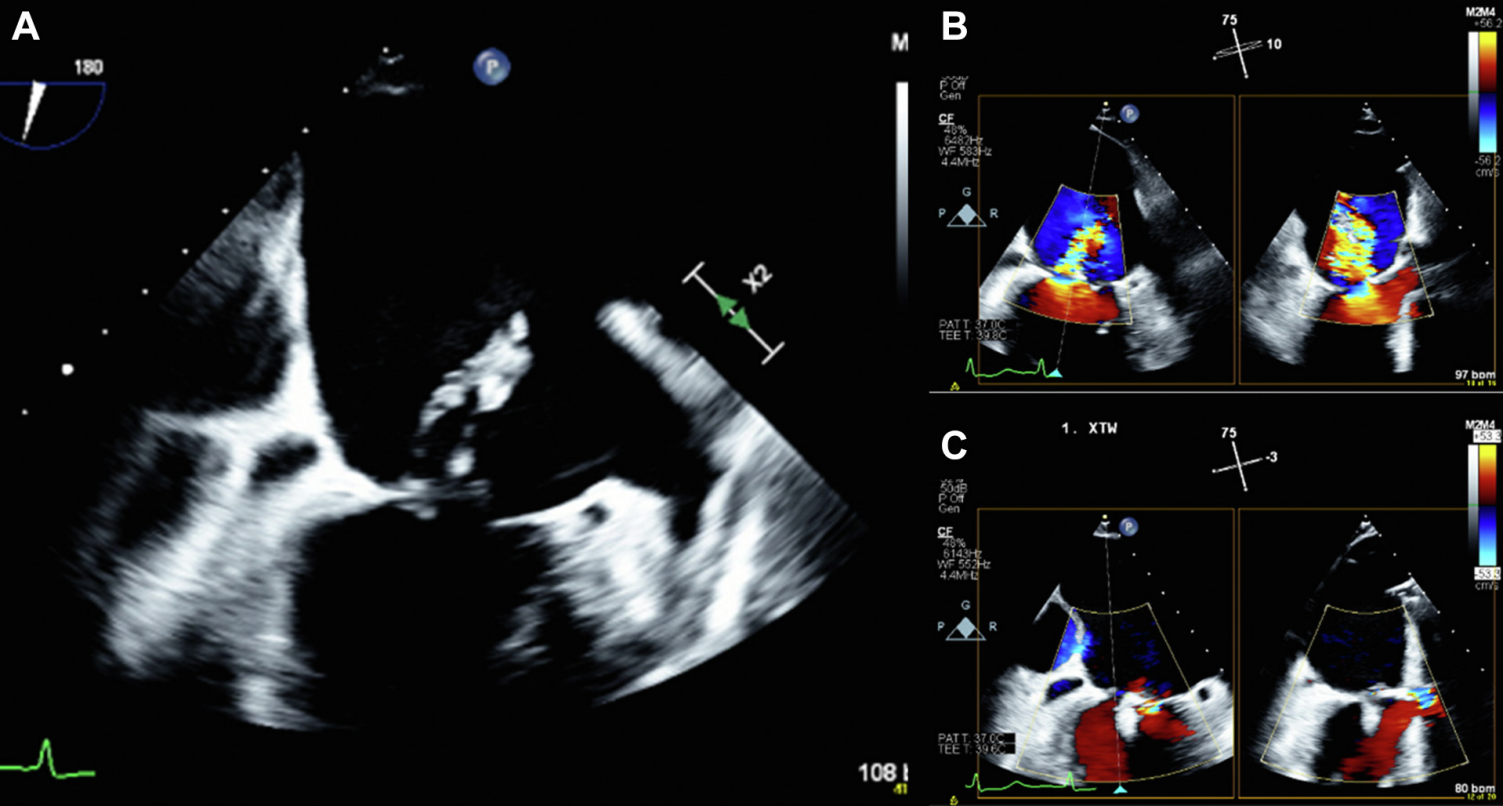
**Postinfarction papillary muscle rupture with acute mitral regurgitation treated with transcatheteredge-to-edgerepair.** (**A**) TEE: complete rupture of the posteromedial papillary muscle. (**B**) TEE: severe mitral regurgitation. (**C**) TEE: postprocedural trace mitral regurgitation. TEE, transesophageal echocardiogram.

After the procedure, the patient required hemodialysis and had signs of aspiration pneumonia. The family decided to withdraw care on postprocedural day 4.

## Discussion

Our case series demonstrated that patients with postinfarction PMR who underwent TEER presented with posteromedial PMR; cardiogenic shock, necessitating mechanical circulatory support; and extreme surgical risk. In all the cases, acute procedural success was achieved, with improved hemodynamic parameters, including left atrial V wave and pulmonary arterial systolic pressures (Table 1). Despite this, survival to discharge was uncommon. Our experience highlights the importance of patient selection.

**Table 1:** Clinical profiles, preprocedural characteristics, intraoperative characteristics, hemodynamic changes, and postprocedural characteristics.

Case	Present case 1	Present case 2	Present case 3	Present case 4	Present case 5	Mean
Patient profile						
Age, y/sex	56/M	68/M	81/F	86/F	84/F	75 ± 12.7
ECG MI	Inferior STEMI	Inferior STEMI	Inferior STEMI	Inferior STEMI	Posteroinferior STEMI	—
Coronary	Mid-RCA	Proximal and mid-RCA	Mid-RCA	—	Proximal R-PDA	—
Culprit PCI	0	+	0	0	+	—
NYHA IV or cardiogenic shock	Cardiogenic shock	Cardiogenic shock	Cardiogenic shock	Cardiogenic shock	Cardiogenic shock	—
LV support	IABP + VA-ECMO	IABP	IABP	IABP	IABP	—
Right ventricular systolic function	Moderately reduced	Moderately reduced	Normal	Mildly reduced	Moderately reduced	—
Logistic EuroSCORE II, %	26.1	27.2	37.3	28.3	39.9	31.8 ± 6.4
STS risk score mortality for MV replacement, %	18.9	28.3	23	24.6	66	32.2 ± 19.2
STS risk score mortality for MV repair, %	18.5	26.4	34	29.4	70	35.7 ± 20.0
Interval between index MI and TEER, d	1	1	0	3	1	1.2 ± 1.1
Interval between PMR and TEER, d	1	0	1	1	1	0.8 ± 0.4
Preprocedural characteristics						
Ruptured papillary muscle	Complete anterolateral	Complete posteromedial	Partial posteromedial	Partial posteromedial	Complete posteromedial	—
Severity of MR	Severe (4+)	Severe (4+)	Severe (4+)	Severe (4+)	Severe (4+)	—
Direction of MR	Posteriorly	Posteriorly	Posteriorly	Posteriorly	Posteriorly	—
Intraoperative characteristics						
MitraClip scallop (system × number of MitraClips)	A2-P2 (NT × 2)	A2-P2 (XTR × 2), A3-P3 (XTR × 1)	A2-P2 (XTR × 1), A3-P3 (NTR × 1)	A2-P2 (XTW × 1), A3-P3 (NT × 1)	A2-P2 (XTW × 1)	2.0 ± 0.7
Device success	+	+	+	+	+	—
Procedure time, min	150	180	180	173	121	160.8 ± 25.4
Hemodynamic changes						
Preprocedural LVEF, %	55	49	82	72	54	62.4 ± 14.0
Postprocedural LVEF, %	35	34	75	33	55	46.4 ± 18.4
Preprocedural LAAP/LAVP (LAMP), mm Hg	35/62 (38)	27/52 (31)	27/34 (22)	11/14 (10)	19/39 (25)	LAVP 40.2 ± 18.3
Postprocedural LAAP/LAVP (LAMP), mm Hg	20/20 (19)	24/24 (20)	31/26 (22)	15/10 (11)	15/15 (14)	LAVP 19.0 ± 6.6
Preprocedural PASP/PADP (PAMP), mm Hg	32/26 (29)	46/18 (27)	26/19 (22)	36/22 (28)	67/40 (52)	PASP 41.4 ± 16.1
Postprocedural PASP/PADP (PAMP), mm Hg	19/13 (15)	40/13 (21)	27/17 (21)	41/20 (27)	45/26 (33)	PASP 34.4 ± 10.9
Preprocedural PAPi	0.3	4	0.9	2.3	1.7	—
Postprocedural PAPi	0.9	2.3	1.4	1.2	1.9	—
Postprocedural characteristics						
MR severity after MitraClip	Moderate (2+)	Mild (1+)	Trace	Trace	Trace	—
MV gradient, mm Hg	0.6	2.8	8.7	2	2.8	—
Outcome						
Hospital outcome	Discharged	Deceased	Deceased	Deceased	Deceased	—
NYHA	I	—	—	—	—	—
Follow-up, mo	1	—	—	—	—	—

ECG, electrocardiogram; F, female; IABP, intra-aortic balloon pump; LAAP, left atrial A wave pressure; LAMP, left atrial mean pressure; LAVP, left atrial V wave pressure; LV, left ventricular; LVEF, left ventricular ejection fraction; M, male; MI, myocardial infarction; MR, mitral regurgitation; MV, mitral valve; NYHA, New York Heart Association; PADP, pulmonary arterial diastolic pressure; PAMP, pulmonary arterial mean pressure; PAPi, pulmonary artery pulsatility index; PASP, pulmonary arterial systolic pressure; PCI, percutaneous coronary intervention; PMR, papillary muscle rupture; R-PDA, right posterior descending artery; RCA, right coronary artery; STEMI, ST-elevation myocardial infarction; STS, Society of Thoracic Surgeons; TEER, transcatheter edge-to-edge repair; VA-ECMO, venoarterial extracorporeal membrane oxygenation.

Acute procedural success was achieved in all 5 of our cases, with successful device implantation and a postimplantation MR grade of ≤2. Urgent TEER was performed in all the patients. The mean interval between PMR and TEER was <24 hours (Table 1). The interval between the diagnosis of MI and TEER was <24 hours in all the patients, except in case 4. The patient did not have PMR until >48 hours after the diagnosis of MI; so, the interval between the diagnosis of MI and TEER was <72 hours. A significant reduction in left atrial V wave height was observed in all the cases (Table 1). Although in cases 3 4, the mean left atrial pressure remained constant after TEER, the patient in case 3 was weaned off inotropes, vasopressors, and IABP the next day, whereas in case 4, the patient remained on inotropes, vasopressors, and IABP in the setting of the *E coli* urinary tract infection. Despite this reduction in MR as well as significant improvement in the pulmonary artery and mean left atrial pressures, only 1 of the 5 patients survived hospitalization.

Mortality was unrelated to the device or procedure. No significant mitral stenosis was introduced by TEER. The postprocedural mitral valve gradient was <5 mm Hg in all the cases despite multiple device implantations, except for the patient in case 3, who had a mitral gradient of 8.7 mm Hg at a heart rate of 83 beats per minute (Table 1). However, the patient’s hemodynamics improved, and she was weaned off inotropes, vasopressors, and IABP the next day. In case 2, the single-leaflet device attachment may have contributed to worsening heart failure; however, the patient had an anoxic brain injury, and care was withdrawn.

In contrast, in a previous report of 5 patients with acute MR due to MI but without PMR, only 1 patient died of multiorgan failure before discharge.^11^ The difference in the outcomes that we observed was likely driven by both the presence of PMR comorbidities associated with PMR, such as older age, female sex, history of heart failure, chronic kidney disease, and delayed presentation. Several additional factors contributed to circulatory shock and the poor outcomes, including bleeding, lower extremity thrombosis, sepsis, renal failure, and aspiration, although these could be interpreted as sequelae of the initial hemodynamic insult. Moreover, the patients in our case series were likely in greater hemodynamic compromise than those in previous reports of acute MR without PMR. Most of the patients in our report had right coronary artery occlusion with probable right ventricular infarction and reduced right ventricular systolic function determined by echocardiograms or pulmonary artery pulsatility index (Table 1). Cases 4 and 5 had significant right-to-left shunt after TEER necessitated closure, and they had the lowest post-TEER mean left atrial pressures, 11 and 14 mm Hg, respectively, in the study (Table 1). The right-to-left shunt may have resulted from the combination of elevated right atrial pressure due to the right ventricular infarction and procedurally reduced left atrial pressure due to TEER. All 5 patients received mechanical circulatory support, mostly IABP. Although the IABP-Shock II trial did not show a mortality benefit from the use of IABPs in patients with MI complicated by cardiogenic shock, the study excluded patients with mechanical complications.^12^ IABP is recommended by guidelines because of its ability to reduce afterload in patients with severe MR due to PMR.^13^ An IREMMI (International REgistry MitraClip in acute Myocardial Infarction) registry-based study of patients with postinfarction acute MR who underwent TEER concluded that cardiogenic shock was not independently associated with mortality.^14^ However, the study lacked statistical power, and the registry included only 6 patients with partial or complete PMR from 18 mostly non-United States centers. A large population analysis revealed a higher in-hospital mortality rate in patients with cardiogenic shock with acute MI who underwent TEER than in those without acute MI (29.6% vs 20.4%, respectively). Age and the use of mechanical circulatory support are strong predictors of in-hospital mortality in the overall population.^15^ Unfortunately, the analysis did not include the number of patients with PMR, procedural characteristics, medication use, hemodynamic parameters, and imaging data.

The 13 cases of PMR treated with TEER that have been published to date are summarized in Table 2. All the patients presented with severe MR; posteromedial PMR was more common than anterolateral PMR, likely because of its single blood supply from the posterior descending artery arising from either the right coronary artery or left circumflex artery.^16^ Nine patients (69%) had the initial clip implanted in the A2-P2 location, and 7 patients (78%) required >1 clip. Although the number of patients was small, this distribution differs substantially from the reported commercial experience of TEER in the United States, in which the A2-P2 segment was addressed in 83%, and >1 clip was used in only 35% of the population with predominantly chronic degenerative MR.^17^ This apparent difference in procedural characteristics is likely the result of anatomic sequelae of PMR, ie, a pathology that needs to be addressed outside of A2-P2 and a broad or wide flail segment that requires multiple clips to improve stability. It is known that TEER in the A2-P2 location is associated with a higher rate of a postimplantation MR grade of ≤2.^18^ Our case of the single-leaflet device attachment involving A3-P3 and the relatively high gradients after the procedure reflect the procedural complexity of TEER for PMR.

**Table 2:** Clinical features, procedural characteristics, and outcome of patients with postinfarction papillary muscle rupture.

Reference, year	Age (y)/sex	NYHA IV/cardiogenic shock	Ruptured papillary muscle	MR severity	LV support	MitraClip scallop (number of MitraClip)	Postimplantation MR severity	Hospital outcome	Follow-up (mo)	Follow-up MR severity	NYHA
Bilge et al,^3^ 2014	60/F	Cardiogenic shock	Anterolateral	Severe (4+)	IABP	A1-P1 (1)	Trace	Discharged	1	Mild (1+)	—
Bilge et al,^8^ 2014	73/M	NYHA IV	Posteromedial	Severe (4+)	None	Posteromedial commissure (1) A2-P2 (1)	Mild (1+)	Discharged	—	—	—
Wolff et al,^2^ 2014	68/M	Cardiogenic shock	Anterolateral	Severe (4+)	IABP	A2-P2 (2)	Moderate (2+)	Discharged	3	Mild-moderate (1-2+)	II
Bahlmann et al,^9^ 2015	77/M	Cardiogenic shock	Complete posteromedial	Severe (4+)	IABP	— (3)	Trace	Discharged	—	—	—
Valle et al,^10^ 2017	84/M	Cardiogenic shock	Partial posteromedial	Severe (4+)	Vasopressor	— (3)	Mild (1+)	Discharged	1	Mild-moderate (1-2+)	II
Papadopoulos et al,^1^ 2019	85/F	Cardiogenic shock	Partial anterolateral	Severe (4+)	IABP	A2-P2 (1) Anterolateral commissure (1)	Mild-moderate	Discharged	20	Moderate (2+)	II
Komatsu et al,^7^ 2019	55/M	Cardiogenic shock	—	Severe (4+)	IABP	A2-P2 (2)	Mild-moderate	Discharged	3	Moderate (2+)	I
Tyler et al,^4^ 2020	51/M	Cardiogenic shock	Anterolateral	Severe (4+)	Impella CP & VV-ECMO	A2-P2 (2)	Mild (1+)	Discharged	6	Mild (1+)	I
Present case 1	56/M	Cardiogenic shock	Complete anterolateral	Severe (4+)	IABP + VA-ECMO	A2-P2 (2)	Moderate (2+)	Discharged	1	—	I
Present case 2	68/M	Cardiogenic shock	Complete posteromedial	Severe (4+)	IABP	A2-P2 (2), A3-P3 (1)	Mild (1+)	Deceased	—	—	—
Present case 3	81/F	Cardiogenic shock	Partial posteromedial	Severe (4+)	IABP	A2-P2 (1), A3-P3 (1)	Trace	Deceased	—	—	—
Present case 4	86/F	Cardiogenic shock	Partial posteromedial	Severe (4+)	IABP	A2-P2 (1), A3-P3 (1)	Trace	Deceased	—	—	—
Present case 5	84/F	Cardiogenic shock	Complete posteromedial	Severe (4+)	IABP	A2-P2 (1)	Trace	Deceased	—	—	—

F, female; IABP, intra-aortic balloon pump; LV, left ventricular; M, male; MR, mitral regurgitation; NYHA, New York Heart Association; VA-ECMO, venoarterial extracorporeal membrane oxygenation; VV-ECMO, venovenous extracorporeal membrane oxygenation.

Transcatheter edge-to-edge repair using MitraClips has been indicated for the treatment of severe symptomatic degenerative MR in patients at prohibitive surgical risk and those with severe symptomatic functional MR despite optimal guideline-directed medical therapy.^5^^,^^6^ TEER has not been approved for acute MR due to MI with or without PMR, which was excluded from the COAPT (Cardiovascular Outcomes Assessment of the MitraClip Percutaneous Therapy for Heart Failure Patients With Functional Mitral Regurgitation) and EVEREST (Endovascular Valve Edge-to-Edge REpair Study) I and II trials.^5^^,^^19^^,^^20^ The current guideline recommends surgical mitral valve replacement for acute severe primary MR, especially in the setting of complete PMR.^13^^,^^21^ However, in reported experiences, only 58% of patients with PMR underwent mitral valve surgery and only 38% of patients with cardiogenic shock due to acute, severe MR were offered mitral valve surgery.^22^^,^^23^ The primary reason for this is the inability to stabilize patients before surgery. This leaves a significant percentage of patients deferred for medical stabilization using vasopressors, inotropes, and mechanical circulatory support. TEER may be a viable therapeutic option given its presumed ability to reduce MR and stabilize PMR by redistributing tension off the ruptured papillary muscle.^10^ Thus, it can serve as a bridge to definite mitral valve surgery. Alternatively, TEER may be considered a destination therapy, as seen in 7 of 9 patients who survived hospitalization and had an MR grade of ≤2 at outpatient follow-up at 20 months (Table 2).

The small numbers of patients from the previously published cases limit the ability to make broad conclusions about patient selection. However, the patients who survived hospitalization appeared to be younger and were less commonly treated with mechanical circulatory support, suggesting less hemodynamic compromise at presentation (Table 2). In our case series, the patient who survived was the youngest (56 years vs a mean of 75 years) at presentation, with the lowest logistic EuroSCORE II (26.1% vs a mean of 31.8%) and lowest Society of Thoracic Surgeons Risk Score for MV repair (18.5% vs a mean of 35.7%), and was treated with prolonged VA-ECMO (Table 1). The proximate cause of mortality after TEER for PMR was sequelae of early hemodynamic decompensation (eg, renal failure, anoxic brain injury, aspiration pneumonia, and sepsis), which suggests that outcomes are better with aggressive support (eg, VA-ECMO) in combination with TEER as soon as possible after PMR is identified.

The current study was small and retrospective in nature. However, the strength of this study is that consecutive patients were treated for postinfarction PMR. The previously reported cases were prone to selective reporting of individual successes. Several generations of the MitraClip device were used in the patients in this case series; only 2 of the 5 patients were treated with a fourth-generation device that allows for independent leaflet grasping and optimization as well as an option for wider clips, and, therefore, our technical outcomes may not reflect what can be achieved with the current technology.

## Conclusion

Transcatheter edge-to-edge repair for acute MR due to postinfarction PMR is associated with a high procedural success rate, but the in-hospital mortality rate remains high. This suggests that early institution of mechanical circulatory support and TEER should be considered to improve survival. Analyzing larger series and registries is necessary for developing best practices using TEER for this rare but fatal mechanical complication of acute MI.

## Declaration of competing interest

Dr Price reports consulting honoraria and/or speaker’s fees from Abbott Vascular, Boston Scientific, W.L. Gore, Medtronic, and InnovHeart. Drs Chang and Romero reported no financial interests.

## Funding sources

This research did not receive any specific grant from funding agencies in the public, commercial, or not-for-profit sectors.

## Ethics statement

This study was conducted in accordance with ethical regulatory requirements and is in full conformity with the Declaration of Helsinki.
